# No Evidence for Moral Reward and Punishment in an Anonymous Context

**DOI:** 10.1371/journal.pone.0150388

**Published:** 2016-03-03

**Authors:** Christine Clavien, Danielle P. Mersch, Michel Chapuisat

**Affiliations:** Department of Ecology and Evolution, University of Lausanne, Lausanne, Switzerland; Mälardalen University, SWEDEN

## Abstract

Human social interactions are regulated by moral norms that define individual obligations and rights. These norms are enforced by punishment of transgressors and reward of followers. Yet, the generality and strength of this drive to punish or reward is unclear, especially when people are not personally involved in the situation and when the actual impact of their sanction is only indirect, i.e., when it diminishes or promotes the social status of the punished or rewarded individual. In a real-life study, we investigated if people are inclined to anonymously punish or reward a person for her past deeds in a different social context. Participants from three socio-professional categories voted anonymously for early career violinists in an important violin competition. We found that participants did not punish an immoral violin candidate, nor did they reward another hyper-moral candidate. On the contrary, one socio-professional category sanctioned hyper-morality. Hence, salient moral information about past behavior did not elicit punishment or reward in an impersonal situation where the impact of the sanction was indirect. We conclude that contextual features play an important role in human motivation to enforce moral norms.

## Introduction

Moral norms are ubiquitous in human societies. They dictate individual obligations and rights in a variety of domains related to harm, care, fairness, reciprocity, loyalty, respect for authority or purity [1]. Moral norms can be enforced by punishing transgressors and rewarding followers [2–5]. Such reward and punishment may contribute to enforce norms in various ways. The most direct way is a stick and carrot education of the punished or rewarded individuals [2]. In a more indirect way, social influence [6,7] and in particular role models [8–10] are known to impact on human norm compliance. Reward and punishment promote or diminish the social status of individuals, and thereby influence who becomes powerful and/or plays a role model in the society. Hence, by punishing immoral individuals and rewarding moral individuals, people may collectively reinforce common moral norms. Punishment and reward can be formal–i.e. executed by an institutionalized authority–or informal–i.e. applied by individual citizens in a decentralized manner [11].

Many studies on human morality assume that humans are intrinsically motivated–i.e. have some built-in inclinations–to enforce the moral norms they adhere to by punishing norm-transgressors and rewarding norm-followers. This assumption can be found in moral philosophy writings [12–14] and in empirical studies in economics [15–17], anthropology [18], social psychology [19–21], or neuroscience [22–24].

However, the generality and strength of this inclination to enforce social norms remain unclear. In particular, do humans perform unconditional moral norm enforcement? By this, we mean do humans have a propensity to punish immoral and reward moral behavior even in the absence of additional non-moral incentives such as prospects for future cooperation, reputation or revenge? And do they also punish and reward past immoral or moral deeds in situations that are not directly linked to these deeds?

Laboratory experiments indicate that norm violation triggers punishment from outside observers–third-party–who are not directly affected by the violation: in many experiments, a fraction of anonymous third-party participants punish uncooperative behavior and reward cooperation at some personal cost, although they could ignore the moral information [15,25–27]. In contrast, no conclusive evidence for costly punishment was found in systematic surveys of anthropological field data [28]. An increasing number of studies indicate that informal moral norm enforcement is conditional: it requires concerns for both norm compliance and personal interests such as direct benefit or reputation [29–35].

To evaluate the generality and strength of the propensity to punish or reward, experimental studies in real-life settings are needed. In a previous study, we assessed whether anonymous judges in a violin competition were inclined to vote against a candidate that was described as immoral–such a vote is a form of punishment because it hinders the career of the candidate [36]. We found that two socio-professional categories–high school and police–ignored the moral information when voting, while a third category–teacher–voted against the immoral candidate.

This result provided evidence against an unconditional inclination to punish immoral behavior and raised three questions. First, did teachers–the only socio-professional category who punished–enforce the moral norm solely because of the immoral character of the candidate, or because of other non-moral consideration? Second, would the three socio-professional categories react differently to other types of immoral behaviors? Third, were most participants reluctant to penalize the immoral violinist because such punishment is a form of harm-doing? Indeed, recent data indicate that humans may be more inclined to enforce moral norms by rewarding moral behavior than by punishing immoral behavior [37,38]. Thus, it is of interest to investigate whether participants are more likely to reward a hyper-moral candidate by voting for her than to penalize an immoral candidate by voting against her.

Here, we perform a novel series of experiments to examine if participants anonymously punish or reward the violinist for a past immoral or moral behavior that she performed in a different social context. We use the same socio-professional categories of participants and the same violin competition setting than in our previous experiment [36], but we vary the content of moral information. The two new scenarios include an immoral and a hyper-moral behavior. These scenarios allow us to examine the extent to which unconditional moral sanctions or rewards are used in an impersonal anonymous situation.

## Material and Methods

### Participants

Participants were students from three types of schools located in French-speaking Switzerland: 1) preparatory schools for future teachers (*n* = 44; 3 classes from 2 schools; 80% female; mean age = 23, SD = 3.97), 2) advanced high schools (*n* = 94; 6 classes from 2 schools; 59% female; mean age = 18, SD = 1.18), and 3) preparatory school for future police officers (*n* = 109; 1 class; 17% female; mean age = 24, SD = 3.31). Two students (one police and one teacher) were discarded from the analysis for reasons detailed below. Our sample size reflects the availability of local classes to participate in the experiment.

### Ethics Statement

Before conducting the experiment in the teacher, police and high schools, we explained the whole test procedure to the relevant authorities and professors and obtained from them, verbal consent and organizational support. By design, throughout the music competition procedure, participants were unaware of contributing to a scientific study. Votes and questionnaires were completed anonymously: neither fellow participants nor experimenters knew for whom individual participants voted. After the experiment, we organized debriefing sessions in which we informed participants of their involvement in a scientific experiment, and asked them for verbal consent to use the data. None of the participants expressed discomfort or asked to withdraw their data from the study and we recorded this information in our test notebook. We did not ask for written consent because we considered that overall, the test procedure was not more invasive than an anonymous opinion survey. This test and consent procedure has been fully approved by our local ethics committee: “Comission cantonale d’éthique de la recherche sur l’être humain”, University of Lausanne.

### Procedure

We used the same cover story as in Clavien *et al*. [36]. Briefly, in place of an ordinary class lesson, participants were involved in the final phase of a music competition. The experimenters, disguised as representatives of a music company, entered the classroom and asked participants to act as music judges in a violin competition (for detailed test procedure and materials, see Clavien *et al*. 2012, supplemental material).

To avoid reputation effects participants were informed in advance that the voting procedure was entirely anonymous. Each participant listened to three recordings of an excerpt from a Mozart Violin Concerto using a computer and headphones. The first recording was from a professional musician (Franco Gulli) and served to familiarize the participants with the music excerpt. The next two recordings were filmed recordings of two pre-selected candidates playing the same Mozart excerpt. Both candidates were female violinists with similar physical appearance and whose faces were blurred. Both were described as finishing students from a European music school. Participants received additional information about the two candidates from short interviews of the candidates’ music professor (see S1 File). The professor provided technical information about the two candidates’ general playing skills, and social information such as opinions about how well each candidate is integrated in her class. Participants were told that the winning candidate would be awarded a record deal, which represents a significant career improvement.

To test whether participant’s voting behavior was influenced by moral information, we designed three treatments (referred to as control, immoral and hyper-moral, respectively) that only differed in the content of the social information about the candidates provided in the professor’s interview (see S1 File). In the control treatment participants (teacher, *n =* 22; high school, *n =* 32; police, *n =* 36) heard neutral information about the social character of each candidate (both candidates are described as nice and normal persons). In the immoral treatment participants (teacher, *n =* 22; high school, *n =* 30; police, *n =* 36) heard that the more talented candidate had a criminal record for selling drugs and still practiced this illicit activity–we hypothesized that participants would categorize such activity as immoral because drug-dealers earn money by selling harming substances–, whereas the less talented candidate was described as a normal person. In the hyper-moral treatment participants (high school, *n =* 32; police, *n =* 37) heard that the less talented candidate was very helpful towards fellow students and provided free music lessons to poor citizens whereas the more talented candidate was described as a normal person. We associated the immoral information to the more talented candidate and the hyper-moral information to the less talented candidate with the aim to generate a conflict between judgment of musical achievement and moral judgment, thus making the decision to punish/reward more salient. We did not test the hyper-moral treatment on the teacher category because we had a smaller number of participants in this socio-professional category.

In all treatments each candidate was randomly associated with one of two sequences of technical information of similar content–i.e. professor’s opinion about candidates’ playing skills. Moreover, because the order in which candidates are presented influences people’s choices [36] we randomized the order in which participants viewed the two candidates in all treatments.

After observing the musical performances and professor interviews, the participants voted for one candidate. Participants were then asked to report their gender, age, what factors played a role in their voting decision (open comment box), and what style of music they listen to regularly: among various proposed choices, they could select “classic”. To check whether participants viewed the important information (music and professor’s interview), the test material included an automatic mouse click tracking system. After all participants had completed the questionnaires we revealed to them that they had participated in an experiment. All but one participants reported to have believed the cover story and were convinced that their vote had an impact on the candidates’ career advancement. The single participant who failed to believe the cover story and reported it in the questionnaire was discarded from the analysis. One additional participant who refused to vote because the professors’ interview would have biased the choice was also discarded.

### Data analysis

To test whether the two types of moral information impact voting behavior overall or differently in the three socio-professional categories, we analyzed separately two datasets: the “immoral dataset” which includes all teacher, high school and police participants allocated to the control and to the immoral treatments and the “hyper-moral dataset” which includes all high school and police participants allocated to the control and to the hyper-moral treatments. For each dataset, we used generalized linear models with a binomial distribution and included as fixed factors the socio-professional category, moral treatment (control, immoral, hyper-moral), technical information about the two candidates’ general playing skills, viewing order of candidates, and interest in classical music. We did not include the gender because it was associated with the socio-professional category (chi-square test of independence, *p* < 0.05), but controlled for the effect of gender in separate analyses (see below). We limited interactions between factors to second order interactions and sequentially simplified the full models (using AIC and chi-square likelihood ratio test for nested models) by removing non-significant effects (*p* > 0.05), beginning with highest-order interactions until we obtained a final model.

In a second series of analyzes, to test for possible effects of gender, we repeated the same procedure separately in each category (teacher, high school and police) for the immoral and hyper-moral treatments. In these subset analyses we included gender, moral treatment, technical information about candidates, viewing order of candidates, and interest in classical music as fixed factors.

To evaluate the risk of a Type II error linked to non-significant results, we investigated with a power test whether our comparison groups (e.g. treatment *versus* control groups, women *versus* men) are sufficiently large to capture 0.2 to 0.25 differences in votes for the best candidate. To calculate the power of our analyses, we used the actual group sizes, we always assumed a medium effect size (h = 0.5) [39], and a significance level of 0.05.

## Results

**Fig 1:**
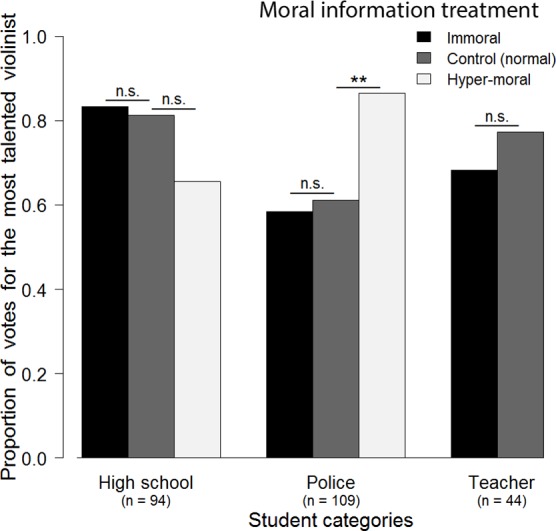
Effect of moral information on third-party observers in a real-life situation. Proportions of votes for the most talented candidate among teacher, high school and police students. Grey bars correspond to the control condition in which both candidates were described as similarly nice, normal persons. Black bars correspond to the immoral treatment condition in which the most talented candidate was described as immoral (she has a criminal record for selling drugs) and the less talented candidate was described as normal. White bars correspond to the hyper-moral treatment in which the most talented candidate was described as normal whereas the less talented candidate was described as hyper-moral (she is a very helpful person and provides free music lessons in poor city areas). The only significant effect of moral information was a tendency to vote against the hyper-moral candidate in the police category. Significance levels (GLM): ** = p < 0.01, n.s. = p > 0.05.

The moral information had very little impact on voting behavior (Fig 1; Tables A & B in S1 File). Participants voted as much for the more talented candidate when she was described as immoral or normal (one-sided power analysis of the “immoral dataset”: power = 0.92). Participants did not favor the less talented candidate when she was described as hyper-moral as compared to normal (one-sided power analysis of the “hyper-moral dataset”: power = 0.88; including the high school subset only: power = 0.52). On the contrary, participants from the police school voted significantly less often (25% less votes) for the hyper-moral candidate than for the candidate described as normal, suggesting punishment of the hyper-moral candidate (GLM on full hyper-moral dataset: interactions between moral information and socio-professional categories: z = -3.02, *p* = 0.003; GLM on police subset of hyper-moral dataset: moral information: z = 2.381, *p* = 0.017).

All participants listened to the music excerpts. Most participants (95% police, 96% teacher, 98% high school) listened to the professors’ interview. We estimate that it takes 12’ to listen once to all the filmed recordings, and about 4’ to complete the questionnaire. Participants’ mean time to complete the task was 18’30. The extra time spent on the task is due to the fact that many participants played the music recordings several times before voting. In total 95% of participants left written comments in the questionnaire explaining why they preferred the candidate they voted for. This indicates that participants were actively involved as violin judges. Only 14% of the comments referred to the social character of the candidates, indicating that most participants did not *consciously* take the social information as a relevant decision factor.

Overall, the participants did not mind receiving information about the candidates’ social character, and they believed this information. The participants could perceive that the professor’s opinion might bias their vote, but only 5 of them (2%) expressed discomfort about this fact in their written comments. None expressed doubts about the veracity of the professor’s information. Participants’ reaction during the debriefing session confirmed this point: they mainly laughed at their own credulity.

In line with previous findings [36], the viewing order affected participants’ judgment (Tables A & B in S1 File). Participants voted more often for the candidate presented second (41% mean increase of votes for the best candidate when presented second; GLM on full immoral dataset: z = -4.938, *p* < 0.001; GLM on full hyper-moral dataset: z = -4.27, *p* < 0.001). This bias was significant in each category of participants (GLM on high school subset of immoral dataset: z = -2.047, *p* = 0.041; high school subset of hyper-moral dataset: z = -2.868, *p* = 0.004; police subset of immoral dataset: z = -3.282, *p* = 0.001; police subset of hyper-moral dataset: z = -3.272, *p* = 0.001; teacher subset of immoral dataset: z = -2.866, *p* = 0.004). Future police officers were less successful at identifying the more talented candidate (GLM immoral dataset: z = 3.016, *p* < 0.003; Fig 1; Table A in S1 File). The other factors we tested for–technical information about candidates (two-sided power analysis of the “immoral dataset”: power = 0.92), interest in classical music (two-sided power analysis of the “immoral dataset”: power = 0.88) and gender (two-sided power analysis including high school dataset: power = 0.66)–had no significant impact on votes.

## Discussion

In our real-life experiment, we found no evidence for a propensity to punish immoral behavior or reward hyper-moral conduct in an anonymous impersonal context. The participants–future teachers, future police officers and high school students–believed that their anonymous vote would impact the career of violinist candidates in a music competition, granting the winner with fame and an increase in social status. Hence, they could have used their vote as an indirect way to enforce moral norms. However the three categories of participants did not penalize the immoral or support the hyper-moral candidate with their votes. To the contrary, police participants disfavored the hyper-moral candidate.

Two features of the situation may contribute to explain why participants did not enforce moral norms in our experiment. First, the reported immoral or hyper-moral behavior had been perpetrated in a social context that was not related to the music competition, making the moral information less salient or relevant. It is thus possible that the participants were able to ignore–or failed to notice–the extraneous information about unrelated activity. Second, participants might have had difficulties in evaluating the personal and social effects of their sanction. Although these two features may contribute to explain the absence of sanction against immoral behavior in our experiment, they did not prevent police participants from voting against the hyper-moral candidate.

We also found that a factor devoid of any musical content influenced participants’ judgment. The viewing order had a strong influence on the voting behavior, with a 41% increase of votes for a candidate when she was viewed second. The impact of viewing order on votes has been documented in other experiments [36,40].

These new results indicate that occasional punishing behavior depends on context. In a previous study, teachers punished the candidate who repeatedly behaved immorally in her music school–for example, she mistuned a colleague’s instrument just before a concert [36]. Here, instead of judging an undisciplined violin student in a school context, teachers had to judge a violin student involved in criminal drug selling perpetrated outside of the school context. We found that teachers tolerated the latter form of immorality. Their contrasted reaction may indicate that teachers view criminal drug selling as morally acceptable. However, it seems more likely that they were influenced by an additional contextual factor when they punished. Teachers may be more motivated to sanction someone who behaves immorally in their usual domain of expertise, possibly because they are trained to teach norms to students or act against students who disturb their working environment.

Overall we found little evidence for punishment or reward in a setting where actors had no personal incentive to intervene and where the sanction reinforced moral norms in an indirect manner, by affecting the social status of the norm follower or transgressor. The high school and police socio-professional categories did not punish the immoral behavior of the violinist whatever the context, while the teachers only punished in the school scenario, but not in the drug-dealing scenario. Overall, the participants did not reward moral behavior or sanction immoral behavior in this particular social setting. It is possible that the participants did not feel legitimate to reward hyper-moral behavior or sanction immoral behaviors that had been performed in a different social context, or that they did not perceive the actual impact of their vote on social norm propagation.

Finally, we tested the plausible hypothesis that participants may be reluctant to penalize the immoral violinist because such punishment is a form of harm-doing. We found that participants did not reward the hyper-moral candidate, and thus were not more likely to reward moral behavior than to penalize immoral behavior. This suggests that the absence of reaction to moral information in our previous experiment was not due to an avoidance of harm-doing [37].

To sum up, our results indicate that humans may be influenced by moral information, but in specific situations. They do not unconditionally punish or reward past immoral or moral behavior performed in a different social context. Additional conditions to salient moral information are needed, such as reputation building [30,35], revenge feelings [41], effect on future interactions [42], professional habitus, or clear opportunity to educate or impact on the behavior of the sanctioned individual.

## Supporting Information

S1 File Translation of professor’s comment and additional statistical information. (PDF)

S2 File Raw data. (XLSX)
