# Supplementary material for: No Evidence for Moral Reward and Punishment in an Anonymous Context
Source: PLoS One. 2016 Mar 3;11(3):e0150388. doi: 10.1371/journal.pone.0150388 (PMC4777527; doi:10.1371/journal.pone.0150388)
Supplement: S1 File — (PDF) [file pone.0150388.s001.pdf]

Supporting Information – S1 File

No evidence for moral reward and punishment in an anonymous context

Christine Clavien, Danielle P. Mersch, Michel Chapuisat

Content

1. Translation of professor's comment

2. Additional tables

1. Translation of professor's comments

In the filmed interviews, the professor first provides technical information about the candidate's general playing skills (i.e. "Technical information"), and then addresses the topic of her social behavior (i.e. "Social information").

Technical information:

In each condition (control, immoral, hyper-moral), the professor describes the more talented and the less talented candidate with alternative scenarios (Type A and B, see below). Specifically, half of the time the more talented candidate was described with the Type A scenario and the less talented with the Type B scenario, and half of the time the more talented candidate was described with the Type B scenario and the less talented candidate with the Type A scenario.

- Type A: "The candidate is very sensitive with her instrument. Her way of playing the violin is rich and full of color. She lives with her instrument. She is able to make her instrument cry and laugh depending on the piece of music she plays. This gives a very human dimension to the music she plays. She may show some stiffness under specific circumstances, but this is something we often see with young players. It will disappear with time."
- Type B: "The candidate has reached a highly professional technical level. She is a virtuoso, worthy of the German or Russian school. She shows stability and handles the bow in a very precise manner. She synchronizes her two hands very well, which enables her to play highly technical pieces of music. Her stability makes her a reliable musician in an orchestra. Sometimes her way of playing lacks a little bit of substance, but this is common among our students. She will easily become a professional musician. It is a promising start."

Social information:

- Candidate described as normal – Type 1: "She is a very friendly person. She is well integrated in the school and in her class. She is a smiling and nice person. There are ups and downs in our school and she proved able to get along with these difficult situations. Difficult situations often occur in a school of arts because we do not only train musicians-as-technicians but also as artists. In these difficult situations, the artist experiences his/her own limits and develops the sensibility that he/she will later use in front of an audience."
- Candidate described as normal – Type 2: "She is a very friendly and pleasant person. She is very studious, always on time and reliable. She also works very well in group and in particular in an orchestra. Our school does not only train artists but also concert violinists and it is important for us that our finishing students are capable of integrating the social microcosms typical of an orchestra. Often the quality of an orchestra also depends on how its component parts interact. Our school proposes courses for learning how to maintain a constructive working atmosphere in groups."
- Candidate described as immoral: "We have had important difficulties with her. All the teachers in the school think the same. She has a dubious lifestyle that is not suitable for her studies. A colleague of mine discussed the problem with her at length, and tried to understand her situation and find a solution… But her repeated absences at school concerts made us discover that she regularly is in trouble with the law, in particular for trafficking illegal substances if you see what I mean. I find it unacceptable. Musical talent does not excuse misbehavior."
- Candidate described as hyper-moral: "In addition to her great musical talent, she is a person of outstanding human qualities. We are very proud of her. For example, our school organizes two charity concerts per year in which she is highly and actively involved as coordinator. The school is also involved in a program for teaching music in the most deprived areas of the town. She is the exclusive coordinator of this program. She is always ready to help and gives the best of herself. She has very touching human qualities which are highly appreciated by the teachers in our school and also by her school mates."

2. Additional tables

Table A

Summary of the results of the final generalized linear model for the immoral dataset (i.e. teacher, high school and police participants allocated to the control and to the immoral treatments). The dependent variable is the vote for the most talented candidate. The reference socio-professional category is the police.

	Factor	Estimate	Std. Error	z value	p value
1	High school category	1.35	0.45	3.02	0.002
2	Teacher category	0.64	0.46	1.40	0.16
3	Candidate viewing order	-2.03	0.46	-4.93	< 0.001

Table B

Summary of the results of the final generalized linear model for the hyper-moral dataset (i.e. high school and police participants allocated to the control and to the hyper-moral treatments). The dependent variable is the vote for the most talented candidate. The reference socio-professional category is the police.

	Factor	Estimate	Std. Error	z value	p value
1	Hyper-moral information	1.32	0.67	1.98	0.048
2	High school category	1.18	0.64	1.84	0.07
3	Candidate viewing order	-2.40	0.56	-4.27	< 0.001
4	Hyper-moral information * High school category	-2.9562	0.9783	-3.022	0.002
